# Improving the Yield and Nutritional Quality of Forage Crops

**DOI:** 10.3389/fpls.2018.00535

**Published:** 2018-04-24

**Authors:** Nicola M. Capstaff, Anthony J. Miller

**Affiliations:** John Innes Centre, Norwich, United Kingdom

**Keywords:** forage, nutritional enhancement, grass production, legumes, breeding, management

## Abstract

Despite being some of the most important crops globally, there has been limited research on forages when compared with cereals, fruits, and vegetables. This review summarizes the literature highlighting the significance of forage crops, the current improvements and some of future directions for improving yield and nutritional quality. We make the point that the knowledge obtained from model plant and grain crops can be applied to forage crops. The timely development of genomics and bioinformatics together with genome editing techniques offer great scope to improve forage crops. Given the social, environmental and economic importance of forage across the globe and especially in poorer countries, this opportunity has enormous potential to improve food security and political stability.

## Introduction

**FIGURE 1:**
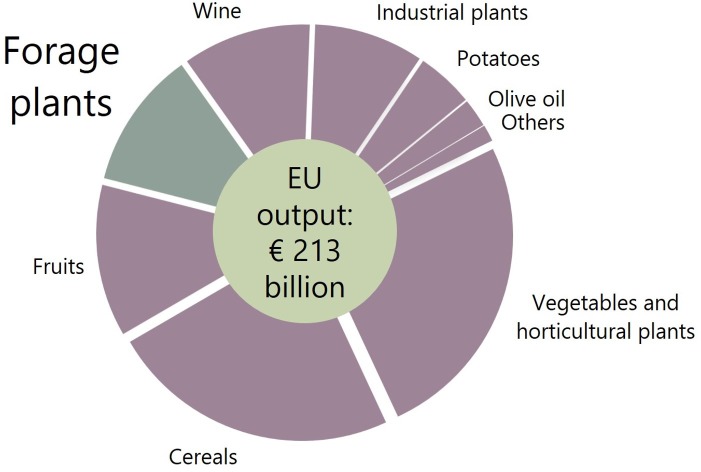
Total European Union output for all crops in 2016 from Eurostat data (Helminger et al., 2016). This includes a substantial portion for forage crops, comparable to the production of fruits and wine.

Forage grasslands are used to feed livestock and globally it has been estimated that they represent 26% of the land area, and 70% of agricultural area (FAO, 2010). Such crops are significant economically, as the European example shows (see **Figure 1**). Forage crops are usually grasses (*Poaceae*) or herbaceous legumes (*Fabaceae*). Some tree legumes such as mulga (*Acacia aneura*) and leadtree (*Leucaena leucocephala*) are also grown in desert and tropical grasslands (Muir et al., 2011). In the tropics, popular grasses include Napier grass (*Pennisetum purpureum*), *Brachiaria*, and *Panicum* species. In the poorest parts of the world livestock production is critically important for smallholders’ livelihoods. Sub-Saharan Africa is an example and frequently women maintain the livestock production systems (Njuki and Sanginga, 2013). In temperate climates, the main grasses include bentgrass (*Agrostis* spp.), fescue (*Festuca* spp.), ryegrass (*Lolium* spp.) and orchard grass (*Dactylis* spp.) or hybrids of these. For example, *Festuca* and *Lolium* hybrids has been developed from 1970s (Ghesquière et al., 2010) giving rise to crops such as *Festulolium pabulare* which combines the superior forage quality of *Lolium multiflorum* with the persistence and stress tolerance of *Festuca arundinacea*. Some maize (*Zea mays*) cultivars have been specifically bred for forage. The commonly cultivated herbaceous legumes are trefoil (*Lotus corniculatus*), medics (*Medicago* spp.), clover (*Trifolium* spp.) and vetches (*Vicia* spp.). *Brassica* forage species include cultivars of oilseed rape (*Brassica napus*) and kale (*Brassica oleracea*). Fodder beet (*Beta vulgaris*) is another temperate forage. The combination of forage crops grown in any country varies depending on climate and livestock needs, however, the perennial legume lucerne or alfalfa (*Medicago* *sativa*) is the most widely cultivated as it can be grown with both temperate and tropical grasses, or as a standalone crop. This is a huge topic to review as there are so many species grown across the world, therefore we have chosen to focus on a few examples, the tropical grasses *Pennisetum* and *Brachiaria*, and more prominently the temperate crops *Lolium* and alfalfa.

In an ideal world, we would all eat pulses rather than the animal products generated from them, as grain legumes are the food that offers the most sustainable future (Foyer et al., 2016). There is continued pressure from many groups to lower human consumption of animal products due to livestock efficiency issues and for human health (Cramer et al., 2017). There is a lack of reliable statistics for the proportion of adults adopting a plant-based diet, but it is estimated to be between 1 and 10% of the population in developed western countries such as within the European and United States (Mcevoy and Woodside, 2010) and studies support these diets as healthy and nutritionally adequate (American Dietetic Association and Dietitians of Canada, 2003). However, the consumption of livestock products can be regarded as important to a healthy diet due to their high nutrient density (CAST, 2013) regardless of the numerous efficiency and environment concerns (Di Paola et al., 2017), particularly true in developing countries where undernourishment incidences are estimated as ∼4–22% of the population (Alexandratos et al., 2006). Livestock production can convert non-edible crops such as the forages into human food, with sustainable intensification possible when inputs and outputs of the system are balanced (Derner et al., 2017).

Moreover, the cultural and social significance of livestock cannot be underestimated and the trend of increased global production is set to continue (Thornton, 2010). Livestock feature prominently across all cultures both in cuisine, but also music and literature. Additionally, in many developing countries the rearing of livestock such as cattle and goats are vital in times of hardship; many view animals as living ‘piggy-banks,’ that can for example pay the family school fees (Herrero et al., 2013). Therefore, in practice livestock production is set to continue throughout the world and forage crops will be grown for coming decades. Plant research has chiefly focussed on grain crops, but here we argue that there is enormous potential for improving forages. Improving the yield and nutritional quality of forage crops can help mitigate the unsustainable negative impacts of livestock production.

## Forage Crops in Livestock Diets

Forage crops can be feed directly to livestock or can be processed by partial drying or pre-digestion. Because of this processing, animal feeds can be categorized as either bulky feeds or concentrates. Bulky feeds are also termed forage and are produced from grass, cereal and legume cropping as described above, such as alfalfa, *Lolium* or a mixture of the two. This forage can be provided to animals directly through grazing pasture land or in a processed form, such as hay (where water content is >15%) or dried (pelleted) biomass. Concentrates are generally cereal, oilseed and legumes seeds, or bi-products of their preparation for human food, biofuel and textile. They can also include high energy feedstuffs such as sugar-rich crop molasses and fats of animal origin, for example fish by-catch discards. In industrialized countries, production of both these categories of feed can surpass the amount produced for plant-based food for human consumption; in United States over double dry matter per-capita per year (DM cap/yr.) is produced for animal feed than for foodstuffs (Krausmann et al., 2008).

Livestock diet can therefore be exclusively forage or largely forage with concentrate supplementation. Concentrate supplementation is used to compensate nutritional deficiencies in the forage supply, increase animal performance such as milk production or at particularly challenging periods of development, for example calving. Due to most livestock diet being of forage this review focuses on the main crops used worldwide and will not discuss concentrates. The amount plant science has contributed to improvements in concentrates has been underappreciated and undervalued in literature, however, the role these crops have on livestock production has been reviewed previously (Erb et al., 2012).

Forage crops can be grown in mixed species cultivation to provide nutritional and environmental benefits. By offering livestock mixed grazing pastures or blending feeds, nutritional quality can be enhanced. For example, alfalfa is the highest-yielding perennial forage legume and produces more protein per unit area than other forage legumes and so can be grown alone or in combination with a range of different grass species. Well-managed alfalfa is normally grown successively for 3 or more years, but if harvested too late in the season the crop cannot survive the winter (Bélanger et al., 2006).

## Forage Nutritional Content

### Digestibility

The nutritional status of a forage crop depends upon the concentration (and ratios) of carbohydrates, proteins, and lipids. The composition of these organic nutrients determines the digestibility (*D*-value) of each crop which along with mineral and vitamins provides the amount of energy which can be derived by the animal (ME measured in MJ/kg DM) (Osbourn, 1980). Such calculations are becoming increasingly prevalent when growers are deciding which crop to grow based and particularly dependent on if the animal is non-ruminant or ruminant.

In forage crops 50–80% of DM is carbohydrate; if this percentage is too low then supplements of grains can be added. The primary types of carbohydrate are the insoluble structural saccharides cellulose and hemicellulose, or the storage forms such as starch and water-soluble polymers (e.g., fructans). These are degraded into simple sugars through cleavage of glycosidic bonds, either by the animal itself (non-ruminant and ruminants) or via microbial digestion and subsequent animal absorption (ruminants only). Different ratios of carbohydrates within the forage crop will have altered downstream digestibility for the animal, especially if the cell-wall structure constrains digestion by the microbial population or limits plant cell wall penetration (Weimer, 1996). Although lignin, a polyphenolic compound within forage, is not a carbohydrate, it has a dramatic impact on the digestibility of cellulose hemicellulose; lignin binds with structural carbohydrates and cell wall proteins and reduces nutrient availability. For forages increased lignin concentration in the growing crop will increase the percentage of indigestible DM. Of the major forage crops grown globally grasses, particularly *Lolium perenne*, have high digestibility due to high soluble sugar content alongside low lignin content (Ruckle et al., 2017).

Animal digestion of simple carbohydrates produces monosaccharides which can be readily metabolized. In ruminants, only microbial digestion of structural carbohydrates produces simple sugars which are subsequently metabolized to pyruvate. Pyruvate is absorbed by the animal and is metabolized further into volatile fatty acids (VFAs) which are a major energy source, (Bergman, 1990). Ruminants absorb VFAs in their rumen, and the rate of this is dependent on the concentration of individual VFAs, rumen pH and the absorptive area in the ruminal lining.

### Protein

Nitrogen (N) availability to animals is predominantly from forage proteins and are estimated using crude total protein Kjeldahl measurements. Protein is usually abundant in the major form of Ribulose-1,5-bisphosphate carboxylase/oxygenase (RuBisCO), although relative amounts vary between species (Wallace et al., 1997). This is especially true when comparing content in grasses with herbaceous legumes, with red clover (*Trifolium pratense*), white clover (*Trifolium repens*) and lucerne (*Medicago sativa*) grown widely due to their high protein value (Ruckle et al., 2017). Again, lignin will severely affect the digestibility of protein. Some micronutrients like proanthocyanidins or condensed tannins also change the digestibility of protein, but they inhibit protein degradation through binding. This can be advantageous as rapid protein degradation is causative of bloat, however, too high a tannin content will mean protein passing through the digestive track is unabsorbed and therefore a loss in nutrition value (Lees, 1992; Piluzza et al., 2014). This means there is a balance between reduced bloat and animal productivity (Mueller-Harvey, 2006). All grasses contain little or no proanthocyanidins, whereas many legumes especially big trefoil (*Lotus pedunculatus*) and Sericea lespedeza (*Lespedeza cuneata*) can have levels as high as 18% DM (Barry and Manley, 1984; Mueller-Harvey, 2006). Other N-containing compounds can be found in forage such as nucleic acids, nitrate and ammonia (Wallace et al., 1997).

### Lipids

Lipids in forage crops are mostly found as polyunsaturated fatty acids (PUFAs) in the range of 10 – 30 g kg^-1^ (Hatfield et al., 2007) of which the most abundant is α-linolenic acid [62% total lipids (Clapham et al., 2005)], with linolenic and palmitic acid also being present (Harfoot and Hazlewood, 1988). These dietary lipids are important in final animal product quality; forage diets with lower PUFA levels than cereal diets can produce leaner meat (Wood et al., 2004; Van Elswyk and McNeill, 2014). Moreover, fresh forage has been shown through numerous studies to produce milk with lowered PUFA content and increased *trans*-fatty acids (Elgersma et al., 2006; Chilliard et al., 2007). Studies have been used to profile PUFAs across forage species, with grasses tending to have more α-linolenic acid when compared to legumes and legumes in turn having higher linolenic acid content (Boufaïed et al., 2003). Striking differences in PUFA content can be seen within species through profiling cultivars, and moreover the harvest period and its environment (Elgersma et al., 2003; Clapham et al., 2005). For example *Lolium perenne*, *Festuca pratensis* (meadow fescue), and *Festulolium* hybrids of the two have been shown to vary not only between species at the beginning of their growth season, but more prominently between individual cutting regimes (Dewhurst et al., 2001).

### Trace Elements

Minerals and trace elements from forages are important for maintaining livestock health. As there is a move toward using fewer antibiotics in animal production the nutritional balance of feed takes on additional importance. Zinc is particularly important for the immune system and supplements can be added to animal feed, but addition of too much results in wasteful excretion, reviewed in Brugger and Windisch (2015). Contrastingly, avoiding accumulation of toxic minerals can also be important for forage crops. Getting the balance right is crucial as low levels of selenium can be beneficial for livestock, but high concentrations are toxic (Zhu et al., 2009). Some elements accumulated in plants can make them unpalatable for livestock, but the ability of forage crops to grow fast and quickly recover from cutting makes them ideal crops for phytoremediation [e.g., Napier grass, (Ishii et al., 2015)].

### Biomass Production

Probably the most important trait of any forage crop is rapid biomass production, as crops are either cut or grazed directly, and nutritional quality depends on the rate of biomass production. Intensive production with faster growth often decreases this nutritional, but this depends on the species grown and some cultivars have better recovery from defoliation. Plant height correlates well with biomass for most crops (e.g., maize) and this factor together with ground area cover are the criteria underpinning methods to assess yields (Freeman et al., 2007).

Many plant species can be grown for forage production, but the ability of the shoot meristem to respond with increased growth after cutting is essential. In some forage species, aboveground grazing or cutting has been correlated with increased root exudation (Paterson and Sim, 1999). This flush of carbon release by roots can stimulate rhizosphere microbes that in turn help to mobilize soil nutrients to sustain aboveground regrowth. Maintaining an optimal nutrient and water supply is very important for forage biomass production. For example, the importance of N supply for re-growth after cutting grass has been demonstrated (Dawson et al., 2004). Furthermore, the previous N status of alfalfa influences its regrowth ability (Meuriot et al., 2004, 2005).

## Improving Forage Crops

### Cultivar Breeding

Due to the relatively recent cultivation of forage crops compared to other agricultural plant species, there were few improvements before 1900. Recently, agricultural trends and the global economic importance of forages, mean new cultivars have been bred. These improvements are helped by many closely related wild populations which can be used in development of new lines (Boller and Green, 2010). The most desirable improvements are increasing dry matter yield (DMY), crop durability and resistance to diseases particularly by pathogenic fungus and pests particularly nematodes, digestibility of DM, and nutritional content of this tissue. Arguably the greatest improvements have been made in breeding of *Medicago* spp., *Trifolium* spp., *Lolium*, and *Festuca*. Large scale breeding programs include testing of these crops, such as NE1010, a multistate cooperative effort of 15 institutes across 12 North-eastern states of United States and Canada (NIMSS, 2017). Similar tropical grass breeding programs include the *Brachiaria* partnership between the International Centre for Tropical Agriculture based in Colombia (C.I.A.T.), the Ugandan National Livestock Resources Research Institute (NaLIRI), the Tanzania Livestock Research Institute (TALIRI), the Institute of Agricultural Research of Mozambique (IIAM) and the Brazilian Agricultural Research Corporation (E.M.B.R.A.P.A.) (CIAT and CGIAR, 2015) which is being conducted across Eastern and Southern Africa.

**FIGURE 2:**
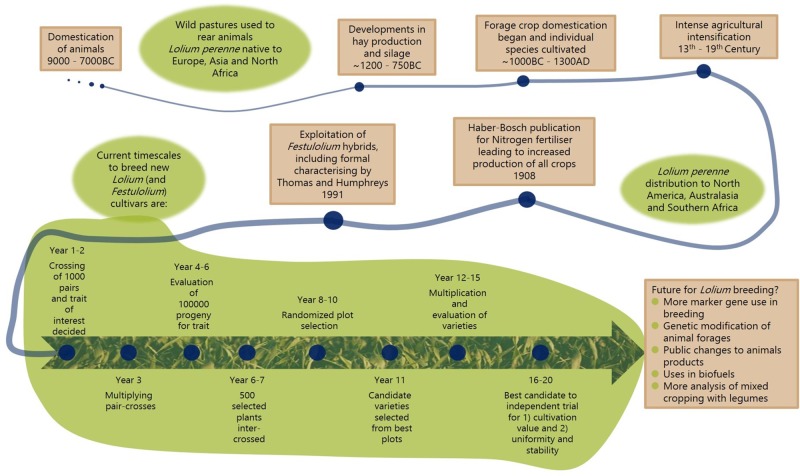
Cultivation timeline of *Lolium perenne* (perennial ryegrass), including current typical breeding regime and future possibilities for breeding programs. The historical timeline is described in brown boxes (Haber, 2002; Hirata, 2011), and general trends in green areas, including modern grass breeding regime as used across Europe (Bruins, 2016).

Breeding programs for forage crops are fraught with difficulties. Individual plants have high genotypic and phenotypic heterogeneity with many species being polyploid, a problem which is exacerbated by in-breeding across many grasses, and few agronomic traits being linked to distinct genes (Poehlman, 1987; Vogel and Pedersen, 1993). Studies have focussed on this problem in specific legumes (Jahufer et al., 2002; Riday and Brummer, 2007; Collins et al., 2012; Luo et al., 2016) and grasses (de Araüjo et al., 2002; Piano et al., 2007; Blackmore et al., 2016). Regardless of these problems there have been some major developments in breeding lines for forages, especially in *Medicago* and *Lolium*. **Figure 2** shows a brief historical timeline of *Lolium* cultivation, and includes the current breeding regimes for grasses; future breeding possibilities are also included and discussed in later sections. One of the most interesting breeding developments is the exploitation of closely related species of *Lolium* and *Festuca* (Thomas and Humphreys, 1991; Humphreys et al., 2003) to create hybrid *Festulolium* cultivars. These cultivars have the high quality characteristics of *Lolium* combined with the stress tolerance and persistence found in *Festuca* (Ghesquière et al., 2010). Backcrossing of *Festulolium* have generated novel hybrids with more stable protein content when compared to parental lines (Humphreys et al., 2014). Advances in phenotyping are making it easier to include the quantification of characteristics in the field; such as high level imaging of growing crops to accurately determine later traits like biomass (Walter et al., 2012).

New cultivars are being helped by advances in sequencing methods that can provide more transcriptomic data (Barrett et al., 2009; Pfeifer et al., 2013; Yates et al., 2014), including the identification of SNPs which may be investigated to improve *Lolium* (Blackmore et al., 2016) and *Trifolium* (Nagy et al., 2013). Draft genomes for such crops and cultivars are becoming increasingly common (Byrne et al., 2015; De Vega et al., 2015; VanBuren et al., 2015) as well as more evidence that model species like *Brachypodium* can direct research (Brkljacic et al., 2011; Rancour et al., 2012). Such research is providing clues to candidate genes which could be used for nutritional enhancement.

### Candidate Genes for Nutritional Enhancement

Identification of potential candidate genes is usually through quantitative trait loci (QTL) analysis or marker-assisted selection (MAS) provided from the above completed genomes. Those identified are studied in relation to biomass and growth traits; in *M. sativa* QTL has been used for lodging resistance and vigor (McCord et al., 2014), plant height and regrowth following harvests in association with *MsaciB* (Robins et al., 2007), candidate gene analysis for flowering and stem height through *CONSTANS-LIKE* (Herrmann et al., 2010) and biochemical markers of ROS resistance genes for drought tolerance correlated to DM (Maghsoodi et al., 2017). The expression of other ROS associated genes of the *Iron-Superoxide Dismutase* family (Myouga et al., 2008) have also been linked to increases in DM in both the legume *M. sativa* (McKersie et al., 2000) and grass *Lolium* cultivars (Warnke et al., 2002).

In *Lolium*, transcriptomics showing differentially expressed genes between wild-type and a dwarf mutant enabled identification of three key genes associated with dwarfism (Li W. et al., 2017), which were subsequently used for forward screens. Markers are used to infer both phenotypic traits and to track inheritance to aid breeding. For instance chloroplast SSRs have been investigated in *Lolium* through a similar technique as above (Diekmann et al., 2012). A thorough re-annotation of the model forage *M. truncatula* genome has also identified hundreds of small, secreted peptides coded by both macronutrient-responsive and nodulation-responsive genes, which could aid reverse genetics for improving many forage crops, especially *M. sativa* (de Bang et al., 2017). Iterative mapping software such as BioMercator (Sosnowski et al., 2012) has been used in *Lolium* to perform meta-QTL analysis using readily available published data (Shinozuka et al., 2012), consequently providing new candidate genes from previous work including orthologs of rice amino acid biosynthesis genes and a marker for reproductive traits, showing how new algorithms can exploit old data. Moreover, BioMercator used to decipher flowering time and height in *M. truncatula* (Julier et al., 2007) directly implicated the above research into *CONSTANS-LIKE* in *M. sativa* (Herrmann et al., 2010; Julier et al., 2010). Such potential ease for transferring model plant knowledge to forage crop research is further discussed below.

**FIGURE 3:**
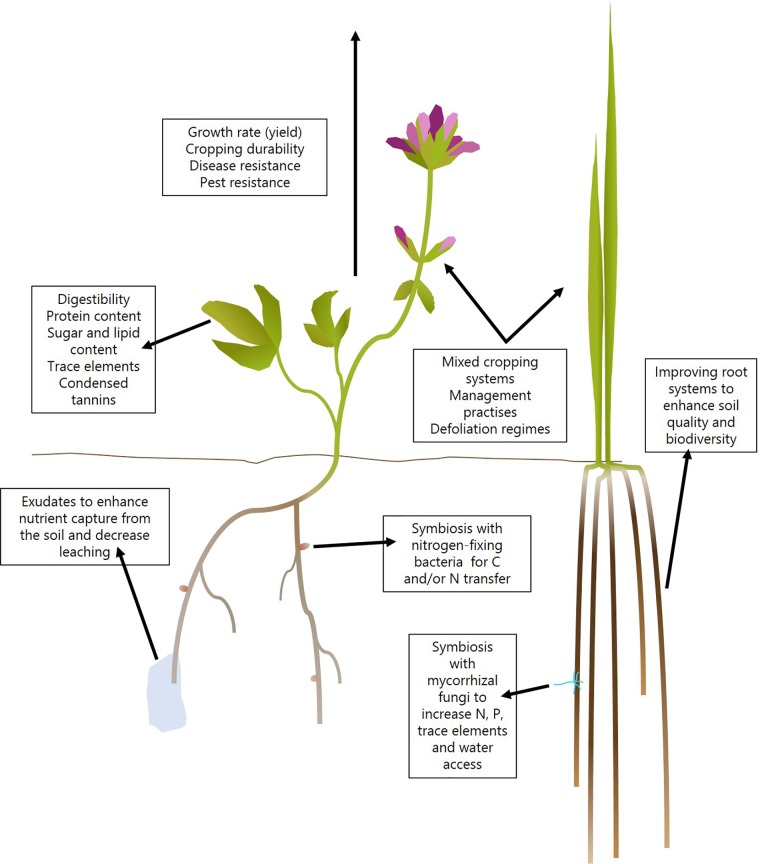
Targets for improved forage crops.

Despite the need to ensure optimal nutritional content especially in the end-product feed, rapid vegetative biomass accumulation is the most desirable trait of a good forage crop, especially those which undergo extensive cutting throughout the growing season. Due to this phenomenon, candidate genes for improving the crops are associated with either photosynthesis or nitrogen use efficiency (NUE). More generally for resistance to biotic and abiotic stresses there is also a huge opportunity for improving traits in forage crops using our genetic knowledge from model plants (see **Figure 3**). **Figure 3** summarizes some of the traits that can be considered for all forage crops.

Such improvements in traits could be aided by achievements in transformation and genetic marker techniques. Reproducible and high efficiency transformation has been developed for temperate cultivars of *Festuca* (Wang and Ge, 2005; Zhang et al., 2006) and *Lolium* (Bajaj et al., 2006; Badenhorst et al., 2016); and more recently for some of the tropical grasses such as *Pennisetum* (Gondo et al., 2017) and *Brachiaria* (Cabral et al., 2015). Some examples of gene editing forage crops to confer stress tolerances have been successful in aiding both biomass increases but also nutritional quality. Transformation of *M. sativa* with the *Arabidopsis Enhanced Drought Tolerance1* gene produced plants with not only increases in root length, shoot height and vegetative biomass, but also increases in proline, soluble sugar and chlorophyll content under drought stress when compared to wild-type (Zheng et al., 2017). Importantly these increases were shown both in the laboratory but also in field conditions. This study also identified the increased expression of many interesting genes, including *M. sativa Heat Shock Protein23* (*HSP23*), a gene already shown to enhance abiotic stress tolerance in both *Nicotiana tabacum* and *Festuca* (Lee et al., 2012a,b) along with other members of the *MsHSP* family (Li et al., 2016; Li Z. et al., 2017). Similarly, for the *Ethylene Response Factor* (*ERF*) family studies have been shown that introducing the *M. sativa* gene into other plants can confer enhanced resistance to salinity; *MsERF9* and *MsERF11* in *Nicotiana* and *Arabidopsis*, respectively (Chen et al., 2012a,b).

### Protein and N Budget

Forage NUE is a target for breeding, particularly as protein content of crops is so valuable. Protein accumulation is linked to N status and when the supply is supra-optimal greater storage occurs. When compared with grain crops that have been bred for high seed starch, forage crops often require N in greater amounts due to their increased growth, storage capacity and higher fiber content (Parsons et al., 1991). For forage crops, it is the leaf tissue biomass that is harvested rather than grains/roots/tubers. Principally NUE for forage crops can be based on N utilization efficiency (NUtE) as we are interested in the highest achievable biomass of the shoot which will form the content to be dried for feed production (Xu et al., 2012). Not only does this include biomass, but also the relative N levels in this tissue; it is not enough to only have a high yield of biomass in the shoots, it must also yield optimal amounts of N. Moreover, when looking at the effect of fertilizer use we are also interested in how both the biomass and N status change on application and thus also N uptake efficiency (NUpE). Forage crops offer challenges for NUE as there is a requirement for optimal yield of shoot biomass with a high N content (NUtE) while also optimizing N fertilizer acquisition (NUpE) throughout the growth season.

**FIGURE 4:**
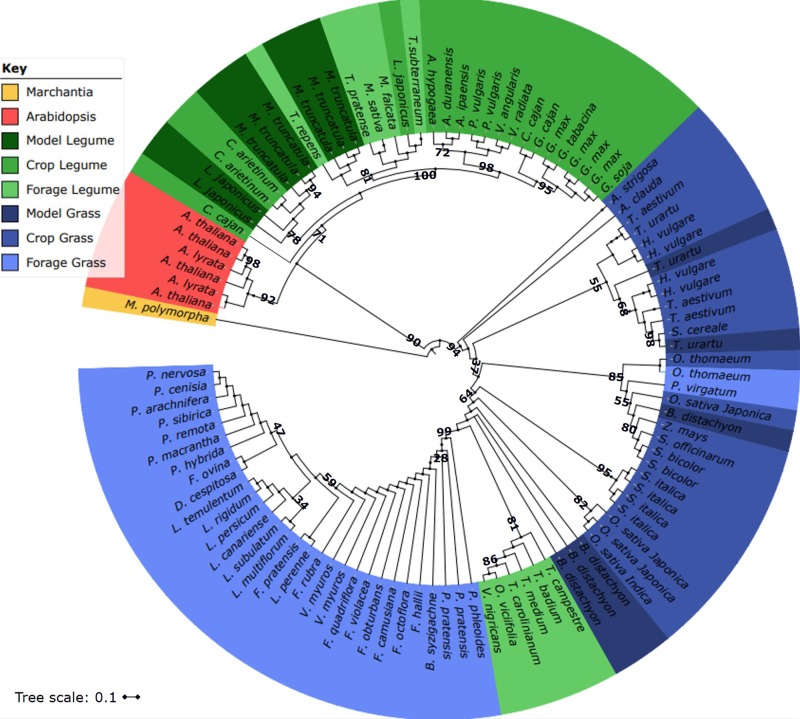
Phylogeny of temperate legumes and grasses for *Ribulose-1,5-bisphosphate carboxylase/oxygenase small subunit* (*rbcS*), also including *Arabidopsis thaliana* and *Marchantia polymorpha* as outliers. Both known and predicted coding sequences for all species of interest were gathered from GenBank, PLAZA 3.0 Dicots and PLAZA 4.0 Monocots. Multiple sequence alignments (MSA) were performed using MAFFT v7 (Katoh and Standley, 2013) with a E-INS-I iterative refinement method and bootstrapping = 100. The phylogeny was built using Newick format in iTOL v3.4.3 (Letunic and Bork, 2007) and a radial phylogenetic tree produced. The tree was color coded to show model species, human food crops and forage crops for both legumes and grasses, although it should be noted that many can overlap in their uses.

As NUE is an important criterion for biomass improvements, many genes relating to N acquisition or metabolism have been the subject of study in model systems. Additionally, genes important in carbon metabolism have also been the focus, due to the links between C:N ratios for plant growth (Jaradat et al., 2009). Despite the long evolutionary divergence between grasses and legumes, many key candidate genes have high genetic similarity, meaning one can use known genes which effect a trait in a forage crop from one species and investigate it within another. For example, a range of vegetative N storage proteins have been identified and the reviewed for leaves (Muntz, 1998) and roots (Bewley, 2002). To illustrate this further, the phylogeny in **Figure 4** is the known and predicted coding sequences for *rbcS* including the model species *Arabidopsis thaliana*, many significant grass and legume crops. Many of the forage crops have high similarity in their coding sequence to more well-studied crop species. For example, *Medicago* and *Trifolium rbcS* sit closely to the legume species which have their genomes sequenced [*Cajanus cajan*, *Cicer arietinum*, *Glycine max*, *Lotus japonicus*, *Medicago truncatula*, and *Phaseolus vulgaris* (Jacob et al., 2016)]. Such sequences can provide a wealth of potential genes of interest for breeding programs (Araújo et al., 2015; Rauf et al., 2016). For example, investigation of *Heat Shock Protein* in *M. truncatula*, found a homologous *HSP70* in *M. sativa* and had a substantial role in stress tolerance when conferred to *A. thaliana* (Li Z. et al., 2017).

In forage crop vegetative biomass, the most important nutrients for livestock are proteins and water-soluble carbohydrates (WSCs), and ideally the post-harvest quality of these should be maintained. There has been considerable interest in developing organ specific proteome reference maps for stems and leaves. The dominant proteins in these tissues are photosynthetic enzymes such as RuBisCO and RuBiCO small unit (rbsS), which for *M. truncatula* make up ∼28.9% of leaf tissue, or other carbon-fixation genes for example glyceraldehyde 3-phosphate dehydrogenase and triose phosphate isomerase, with structural protein such as lignin biosynthesis being more concentrated in stems (Watson et al., 2003). As the *D*-value of forage is mostly linked to cell wall concentration and a reduction of this can aid digestibility (Jung and Allen, 1995; Jung et al., 2012), some proteomes have looked even more specifically at such tissues (Gokulakannan and Niehaus, 2010).

Some research has focussed on transgenic approaches to increase and enhance amino acids and proteins. As many forages have low concentrations of the sulfur-containing amino acids of methionine and cysteine, both important in animal and human nutrition (Ball et al., 2006), some studies have specifically aimed at increasing these levels by over-expression. These have included using lupins (*Lupinus albus*) (Molvig et al., 1997; Tabe et al., 2010) and soybean (Dinkins et al., 2001; Tabe and Droux, 2002), used as forage sources.

Apart from cultivar differences which can be improved with breeding programs or specific transgenic approaches, the most significant changes in nutritional content is due to stresses (Araújo et al., 2015). Consequently, stress proteomes have also been used for vegetative tissue; lupin stem proteins have been analyzed under water stress to show increases in serine protease and cysteine protease required for remobilization of proteins (Pinheiro et al., 2005); in grasspea (*Lathyrus sativus*) seedlings under either salinity, low temperature or ABA stress gave rise to the identification of 48 stress-responsive proteins (SRPs) which include those important dominant proteins discussed above (Chattopadhyay et al., 2011); in *M. sativa* drought conditions showed remobilization of RuBisCO-derived N could compensate for the decreases in N assimilation (Aranjuelo et al., 2011). Moreover, through harvesting regimes, forage crops undergo extreme stress which has shown to cause the remobilization of vegetative storage proteins (VSPs) to boost new shoot regrowth in both *Medicago* and *Trifolium* as well as being important for cultivars with better cold tolerance (Avice et al., 2003), whereas *Lolium* has shown how defoliation increases the relative proportions of certain proteins, particularly asparagine and glutamine (Bigot et al., 1991).

Finally, the N consumed by livestock is recycled and increasing ruminant productivity is a major target for as the conversion of plant to microbial protein is inefficient. It was estimated that as much as 70% of the plant N eaten by animals for milk or meat production is excreted as ammonia or urea to the environment (MacRae and Ulyatt, 1974; Kingston-Smith et al., 2008; Kingston-Smith et al., 2010). Furthermore, the process of rumen fermentation is important for the generation of greenhouse gasses like methane (Bannink et al., 2008; Dijkstra et al., 2011).

### Rhizosphere Microbiome

The impact of genomics extends beyond the crop plants to their environmental interface. For example, the rhizosphere microbiome is likely to be a future target for improving the nutritional quality of crops. Epiphytic bacteria living on and in the plant, may be important for crop health and nutrition, and some microorganisms can fix atmospheric N within legume root systems. Bacteria living with plants may be able to assist in digestion and absorption of forage eaten by livestock. These bacteria may improve the uptake of trace elements in the animal gut by the production of specific binding molecules and/or siderophores. In the soil, the rhizosphere microbiome is important for nutrient cycling and uptake, particularly in low input systems like those grown in the tropics. The inoculation of new forage crops with beneficial microorganisms is likely to be a target for research and use in future crops, coupled with rhizosphere microbiome research of root exudate composition.

The root is known to directly modify the rhizosphere population by altering the chemical constituents of root exudates. For example, the roots of the tropical grass *Brachiaria* specifically produce a chemical shown to inhibit nitrifying bacteria and to specifically block ammonia-oxidizing pathways in soil bacteria, the first step in the process of converting ammonium to nitrate (Byrnes et al., 2017). Soil ammonia-oxidizing bacteria quickly convert urea or NH_4_^+^ fertilizer to NO_3_^-^. Soil N form is fundamental for crop acquisition, as NO_3_^-^ is mobile and readily leached while NH_4_^+^ binds. Nitrification inhibitors have been identified in root exudates from several legumes and grasses including sorghum and rice, but by far the largest activity was detected in the tropical grass *Brachiaria humidicola* (Subbarao et al., 2009). In rice, the ability of root exudates to inhibit nitrification varied between cultivars from 5 to 50%, but was not significantly higher in three ancestral lines (Tanaka et al., 2010). The biological nitrification inhibitor (BNI) activity of root exudates has been assayed using a recombinant luminescent reporter ammonia-oxidizing bacteria *Nitrosomonas europaea* (Subbarao et al., 2006). In *Brachiaria*, roots exudate the cyclic diterpene “brachialactone,” (Subbarao et al., 2009); brachialactone has a 5-8-5-membered ring system and a γ-lactone ring and contributed to 60–90% of the BNI activity released from the roots of this tropical grass. This exciting example offers the potential for transferring this trait to other forage crops to improve NUE. In the future synthetic pathways to produce plant nitrification inhibitors will be fully elucidated, providing the opportunity to capture this trait in forages and transfer to other crops to improve yield and nitrogen acquisition.

### Digestibility

As protein digestion and uptake in livestock is directly related to energy availability (ME) (Nocek and Russell, 1988; McCarthy et al., 1989) it is important to increase WSC in many forage crops, especially grasses (Miller et al., 2001). In *Lolium* WSCs include fructans which are the most important storage polysaccharide and thus improved metabolism of fructan from sucrose can help improve the *D*-value (Chalmers et al., 2005). Use of distinct *Fructan:Fructan 6G-fructosyltransferase* sequence variants has shown to increase fructan levels at warmer temperatures in *Lolium*, thus hoping to aid development of high sugar-content grasses even at changing climates (Rasmussen et al., 2014). The amounts of WSCs are strongly associated with the N availability to the root (Roche et al., 2017) highlighting the importance of C:N balance in vegetative tissue (Louahlia et al., 2008). Furthermore, the amounts of WSCs varies between varieties as well as within the environment; *Lolium* cultivars AberMagic, AberDart, and AberElite all had highest growth rates correlated to highest WSC concentration during spring/summer, corresponding to high N availability from the roots alongside optimal photosynthesis conditions (Winters et al., 2010). Recent advances in the identification and manipulation of photosynthesis promoters for both *Lolium perenne RBCS* and *Chlorophyll a/b Binding* (*CAB*) (Panter et al., 2017) has provided transgenic lines for assessing increases in yield, fiber and, more importantly for digestibility, the fructan concentrations in both pseudostem and leaf blades in field trials (Badenhorst et al., 2018). Such work provides a platform for future studies to identify promotors important in other nutritional traits.

The amounts of resistant starch are important for the digestibility and nutritional content of forage crops. Resistant starch (RS) generally has lower digestibility until it reaches the large intestine (Englyst et al., 1999), where in ruminants more digestion can occur (Raigond et al., 2015). Research studies have shown that *M. sativa* has advantages as a feed source over cereals for enhanced *D*-value (Giuberti et al., 2018). One major difference between dietary RS is that it is seen to have advantages in the human diet by providing more fiber, but disadvantages in livestock feed for non-ruminants as it remains undigested. In general, lower RS will improve the digestibility of forage crops for both ruminant and non-ruminant livestock. As a crops *D*-value is closely linked with its starch, protein and lignin content, genomic studies have begun large-scale genome-wide association studies (GWAS) to confirm correlations across a range of traits, such as using three distinct alfalfa cultivars with a high-throughput genotyping-by-sequencing approach (Biazzi et al., 2017). However, this study did highlight that differences in SNPs associated in different tissue types (shoots and leaves) can vary in correlation with traits such as protein content, and so care must be taken when using GWAS to aid crop improvements.

Another substantial nutrient in forage crops is that of proanthocyanidins or condensed tannins (CTs). CTs bind to protein making it unavailable to digestion for ruminants until it reaches the rumen, and thusly an important trait in increasing the *D*-value of a crop (Min et al., 2003), although too high a CT content can be harmful restricting fermentation, especially in low leaf protein content species. A compromise is therefore desirable, with the moderate CT of 2–4% of the forage biomass giving the optimal *D*-value (Dixon et al., 2005). Whilst some species of legumes have optimum levels of CTs such as *Lotus corniculatus*, others such as *Onobrychis viciifolia* and *Trifolium ambiguum* are often poor choices for forage in many climates (Min et al., 2003; Baker, 2012); which means there is more scope to increase CTs concentrations in high yielding species where they are low such as *M. sativa* and *Trifolium repens* rather than increase growth traits aforementioned (Burggraaf et al., 2006; Salunkhe et al., 2017).

As the CT synthesis pathway has been well-characterized in *Arabidopsis* with the transcriptional regulators R2R3 MYB, bHLH, and WD40 protein identified as having a central role in final CT content (Lepiniec et al., 2006). Such knowledge can be used to manipulate forage crops. The *R2R3 MYB* homolog *MtPAR* in the *M. truncatula* seed coat has been characterized and hairy root transformation in alfalfa resulted in the accumulation of CTs to the level of ∼20 mg/g shoot biomass (Verdier et al., 2012), although this is still below the desirable concentration. A similar study showed that expression of the *TaMYB14* transcription factor from a low-yielding forage activates CT biosynthesis in both *Trifolium* and *Medicago* (Hancock et al., 2012). Other approaches have involved characterizing early steps of CT biosynthesis in *M. truncatula* in the hope to later target crop relatives (Pang et al., 2007), whilst others have looked at how relative amounts of CT differ between leaves and higher concentration containing flowers to see if changing flowering in *Trifolium* could improve its *D*-value (Burggraaf et al., 2008). There has been an effort to engineer better digestibility in some forage cultivars (Wang and Brummer, 2012) and microbial pre-digestion after cutting and before feeding, including microbial supplements (Boyd et al., 2011; West and Bernard, 2011; Elghandour et al., 2015), can be used to enhance this.

### Biomass Production

As biomass yield is the main target for forage crop improvement, more rapidly growing cultivars can be targeted for breeding. Studies have consequently focussed on heading date (Fe et al., 2015) and flowering time regulation (Skøt et al., 2011; Shinozuka et al., 2012) in *Lolium* by developing Genomic Prediction models and QTL mapping as described previously (Nuñez and Yamada, 2017). Manipulating genes involved in delayed senescence has been targeted for increasing biomass yields. The introduction of the 5′ flanking region of the *Zea mays* cysteine protease gene *SEE1* in *Lolium multiflorum* has shown this promoter region to increase leaf lifespan by approximately 8–16 days (Li et al., 2004). A similar study using the *Arabidopsis Senescence-Associated Gene12* (*SAG12*) promotor also delayed senescence in *M. sativa* with notable chlorophyll and yield increases even after 3 months of growth (Calderini et al., 2007). A final example is the expression of the *Panicum virgatum NAC1* and *NAC2* transcription factors in *Arabidopsis atnap* lines (mutants with defective senescence) to restore wild-type phenotype, predominantly measured using total chlorophyll concentrations (Yang et al., 2015).

However, fast growth must also be coupled with the ability of the plants to respond to cutting by providing rapid regrowth. Growth rates and recovery from cutting are traits that are relatively easy to select for in breeding trials, and have been of regular interest to researchers for many decades in both *Lolium* and *Medicago* (Wilman et al., 1977; Vance et al., 1979). In addition, cutting experiments using 13C and 15N in both *Lolium* and *Medicago* have shown how the soil is affected both for dissolved organic C and N, and microbial biomass, demonstrating that management schemes can be critical to subsequent soil health (Schmitt et al., 2013). An ability to rapidly regrow may increase the susceptibility of the plant to insect and pathogens and this is worthy of further investigation. The relationship between tissue wounding and plant immunity is a topic that is quickly developing and there is now good evidence that tissue growth rate is closely linked with immunity (Huot et al., 2014).

Thusly, management schemes for forage crops are very important for yield. For example, choosing when to cut or graze a crop is crucial for subsequent regrowth of the plant (Karn et al., 2006; Asaadi and Yazdi, 2011; Bumb et al., 2016). To assist in this choice there is scope for the use of molecular markers, with the future possibility of a PCR test for the optimal time harvest based on the expression of candidate genes like storage proteins. Such tissue testing of crops can also be used for decisions on the timing of fertilizer applications as the two evaluations are made at around the same time. There is scope to identify a suite of marker genes that can be used to help decide when these key decisions are made.

Mixed cropping schemes are already widely used for forage crops and there are clear advantages in growing legumes and grasses together. Legumes increase soil N through their N fixation symbiosis with *Rhizobium*, with their biological nitrogen fixation ranging from 32 to 115 kg ha^-1^ (Iannetta et al., 2016). This can in turn decrease subsequent fertilizer use for crops grown thereafter, a reduction between 23 and 31 kg N ha^-1^ (Preissel et al., 2015). Numerous intercropping regimes have been tested including modeling of various climatic and soil texture parameters (Bachinger and Reining, 2009). Transfer of N from legume to crop, including in grasslands, has been investigated (Pirhofer-Walzl et al., 2012). However, it is still unknown how this interaction affects N movement and leaching through the soil profile. Such an investigation is required to give evidence of environment changes as well as crop productivity. Mixed species cultivation also has advantages for disease and extreme weather resistance as the susceptibility of the plants to these stresses varies between cultivars and species. Forage breeding has focussed on monoculture selection regimes and there is scope for better mixed species crops that could be included in trials for new varieties. Some advantages and disadvantages of mixed forage crops are summarized below in **Table 1**.

**Table 1:** The advantages and disadvantages of growing forage crops in mixed systems.

Mixed cropping	
Advantages	Disadvantages
Soil nutrient availability-each species may have different strategies to mobilize nutrients	Grow rates and optimal harvest date can differ
Pathogen and pest susceptibility is different	Specialist equipment may be needed
Legume can supply N	Competition for resources
Stem support in canopy	One species may host pathogens
Root depth for water access and improved soil structure	Monitoring more than one species at a time to keep up with needs

Growing forage crops for improved nutritional quality has not been a target for breeding programs, rather yield and climate tolerance have been the drivers. Future crops must be tolerant of climate changes and weather extremes. Unlike many crops where monocropping is most productive, forage crops have the advantage that they can be easily grown in combination without lowering productivity. Such a trend has been shown across multiple trials as well as increasing biodiversity (Tilman et al., 2001, 2006; Weigelt et al., 2009). As with any system that promotes biodiversity whilst still being productive, this can mean not only lowered costs to manage but also help cultural agriculture acceptability, with consumers becoming more aware of the effect the production of their food has environmentally (Scherr and McNeely, 2008).

### Trace Elements

Plant research has focussed on the goal of biofortifying cereals, but there is also potential to improve the nutritional quality of forage crops. The economic importance of livestock production in the poorest parts of the world offers the opportunity to biofortify animal crops thereby improving the health of these animals and both directly and indirectly their owners. The knowledge base developed for grain biofortification (e.g., candidate plant metal transporters) has yet to be applied to forage crops. For example, transporter proteins for iron and zinc storage have been identified in cereals (Connorton et al., 2017; Menguer et al., 2017) and their equivalents in forages have yet to be identified.

Although very abundant in most soils, silicon is particularly required by grasses (Tubana et al., 2016) and is therefore likely to be important for the optimal growth of many forage crops. Silicon is important for cell wall structure and therefore resistance to pathogens and pests, however, it may have a negative impact on digestibility. The supply of this nutrient may become limiting for forage crops, particularly as the plant biomass is regularly removed from the field and silicon is not yet a routine addition to fertilizer.

Most species of forage crops can form mycorrhizal associations and this type of symbiosis is important for acquisition of trace elements. For natural grazing, these symbiotic associations are particularly important, but when fertilizer is added to cultivated forage crops mycorrhiza are suppressed (MacLean et al., 2017). Enhancing this symbiosis by inoculation of forage crops with mycorrhizal fungi has the potential to improve the mineral element composition of the feed. The fungal symbiosis has additional benefits for the plant by increasing the soil area mined for nutrients and water; this can be crucial during extreme weather events such as drought. Furthermore, a balanced and optimized root rhizosphere microbiome is essential for optimal root function and this applies to all crops including forage (Mommer et al., 2016).

### Environmental Footprint of Forage Crops

As in all agriculture, improving water and nutrient use efficiency is a target for forage crops. The general fertilizer requirement of maize grown for forage and for grain are the same as that for a biomass crop. N requirements differ greatly for forage crops, and legumes and rhizome crops like *Miscanthus* have low N requirements (Dierking et al., 2017). Improving NUE using transporter marker genes as indicators of the crop status in the field could be valuable (Fan et al., 2017). Targeting particularly the NUpE component of NUE is important for minimizing the wasteful and environmentally damaging losses of excess N fertilizer additions.

As discussed above for protein content, biomass production and cutting/grazing decisions there is the potential to develop gene markers that can indicate the N status of each type of forage crop. Mixed plant communities tend to have better NUE, probably because each species has a different temporal pattern of N uptake, resulting from different growth rates and root architecture (Tilman et al., 2001; Weigelt et al., 2009). In more affluent countries the relatively low chemical fertilizer prices do not encourage more judicious use of fertilizer for forage crops, but the threat of legislation for overuse has provided a new incentive for better fertilizer use efficiency. There is plenty of scope for improving the NUE of forage crops particularly as breeding programs have not focussed on this trail. For water acquisition, the long tap roots of *Medicago* are ideal for penetrating deep for water and nutrients. Varietal differences in this important trait have long been known (McIntosh and Miller, 1980) and the choice of cultivar depends on the soil type, climate and cropping regime that is required.

## Conclusions and Future Directions

### Future Performance Improvements Using Genomics

The availability of genomics and bioinformatics has revolutionized all biology and as databases expand to include more species and cultivars this information can assist forage breeders to improve crop performance. The future possibilities for breeding of forage crops using *Lolium* as an example are shown in **Figure 2**. By comparing cultivar sequence information and using GWAS for traits such as high vegetative tissue concentrations of protein, NUpE or specific trace elements the nutritional quality and yield of forage crops can be improved. Some SNPs in key genes that have been identified in model plants can be the targets for gene editing techniques (Bonhomme et al., 2014; Slavov et al., 2014; Thorogood et al., 2017). TILLING lines are also being used in many forage crops to study gene function (Carelli et al., 2013; Dalmais et al., 2013; Manzanares et al., 2016). Furthermore, as shown with the *rbcS* example in **Figure 4**, sequence information can be used for the design of PCR primers which can be used for tissue testing. These tests can be used to rapidly identify general health and nutritional status of crops as well as specific pathogens. One bottleneck is likely to be the transfer of the new genetic information into forage crops. For example, GM forage crops may be more acceptable to the public, as if fed to animals their entry into the human food chain is indirect. The use of CRISPR/Cas9 technology may provide an acceptable route for such manipulations, and as with many crops such feasibility studies have begun in forage crops; the mutation of the *Medicago sativa Squamosa Promoter Binding Protein Like9* (*SPL9*) has been attempted and validated (Gao et al., 2018), although poor genome editing efficiency is limiting advances at present. Many candidate genes have been identified which may be quickly transferred into forage crops, but the technology for transformation is limiting development of these improved plants. In the future genome editing may become more accepted, particularly perhaps for animal feed crops.

### Focusing on Roots

As discussed above high-yield, low-input vegetative biomass is desired for forage crop production. This has meant aboveground phenotyping strategies are being widely developed using predominantly imaging and spectral data (Walter et al., 2012), although more research is needed to see how vegetative phenotyping will work across different species, especially in mixed-cropping systems. However, although the need for well-developed, established root systems is clearly important (Kell, 2011; Nacry et al., 2013), breeding for belowground traits has been largely disregarded. This is unsurprising as with all crops, root phenotyping is difficult, being hidden in the soil and therefore labor intensive and difficult to sample. Any current root system improvements have been the consequence of vegetative drought and salinity assays discussed previously.

Consequently, there has been a shift of focus toward breeding for underground traits in forage crops; across plant science this has been termed the next green revolution step (Lynch, 2007; Den Herder et al., 2010). Before phenotyping can even begin it is necessary to determine which kind of improvements are necessary, of which 2 main categories are found. The first is to improve root systems for the plant itself. This could include increasing fine root biomass, lateral root initiation, or in the case of legumes nodulation by *Sinorhizobium*, for increased nutrient uptake (Jackson et al., 1997; Ariel et al., 2010; Downie, 2010; Wang et al., 2010), or instead increasing root density or taproot length for either nutrient and/or water uptake, or resilience to stress such as defoliation (Dawson et al., 2004; Erice et al., 2007; Ghesquière et al., 2010; Kell, 2011).

The second category is the improvement of root systems to aid the environment. This target is to improve agricultural land not just for production but also in terms of the ecosystem services, and this is especially true in the case of forage crops (Marshall et al., 2016). Forages and grasslands can provide ecosystem services that are wide-ranging and highly linked to root function including soil C-sequestration important for climate change (Kell, 2011, 2012), or lowering run-off of land thus helping to lessen flooding and soil erosion (Macleod et al., 2013). The idea of using both non-leguminous and leguminous forage crops as cover crops to mitigate climate change is gaining appreciation, (Kaye and Quemada, 2017). Another point to note is that many perennial grasses including *Miscanthus* and *Panicum* can be used for biofuel production but the characteristics required for a forage crop do not always match with those of a biofuel (Yang and Udvardi, 2018), although efficient root function and structure is likely to be a characteristic desirable for both agricultural sectors.

Whether to improve plant performance or that of the environment, advances in phenotyping root systems will be crucial, including characterizing the plasticity of the system whilst the plant is growing. At present there are a plethora of root analysis software available (Paez-Garcia et al., 2015), but these require imaging roots either grown artificially such as on plates or already taken from the field and therefore evasive. There is therefore an increased interest in developing imaging techniques of plants grown in clear media to chart phenotypic changes throughout growth, or more promisingly the use of X-ray computed tomography (CT) scanning to give high resolution 3D models of the growing root system (Zhu et al., 2011).

### Developing Management Systems

At present forage growers cannot easily and reliably determine the N status of their crops. For maximum biomass production, it is important to maintain the N status of the crop throughout the growing season and this requires an optimized soil N supply (Hofer et al., 2017). Application of too much N fertilizer results in wasteful run-off and sub-optimal supply results in decreased biomass production. Studies have already shown, through ^15^N labeling *of Lolium*, how deficiency caused by low N fertilizer application causes an increase in the protein substrate pool whereas the store pool decreased in size and turnover rate (Lehmeier et al., 2013). This highlights the importance of fertilizer studies for N composition of forage crop vegetative tissue. Maintaining N supply for maximal yield is limited by two factors: (1) unreliable and unreproducible tests for soil N levels (Knight, 2006) and (2) an easy reliable measure of the crop’s status.

Presently farmers take limited samples across their growing area in the hope that this is representative of the N in the whole plot through the growing season. Nevertheless, this does not indicate a plant’s N status or provide a measure of NUE. Some research has focused on the use of spectral data to evaluate crop efficiency (Foster et al., 2017), but such techniques require further investigation and can give false readings caused by pathogen attack. Sensors for N contents of soil are also being developed, however, these can be a costly solution (Shaw et al., 2016). Due to these problems, it may be better if the farmer could determine the crop N status directly and then make a more informed decision as to how they should subsequently fertilize the plot. This would enable more efficient fertilizer use, thus increasing forage biomass with lowered costs. Furthermore, for forage that includes legumes these N budget problems are complicated by the additional input of gaseous N-fixation. Other strategies of crop testing should be developed to reliably inform the grower of NUE efficiency.

### Final Animal Product Studies

As forages are grown to rear livestock which in turn becomes food products for humans it is also important to view research in plant science from a livestock study prospective, of which has been touched upon above when discussing nutritional composition of crops. At present many countries adopt large-scale, concentrate-feeding led livestock production like that of the United States, with many potential human health risks due to bacteria, antibiotic-resistant bacteria, prion, and dioxin presence in end products (Sapkota et al., 2007). Despite a rise in concentrate-feeding, forage crops are still used widely as the main source of feed due to its high-yields of DM and energy for low costs (Reynolds, 2000), although usually studies focus on investigating a combination of both especially at various stages of development. For example, studies comparing growth of cattle fed a grass-diet instead of a linseed diet found the end product meat had a healthier fatty acid profile high in beneficial n-3 PUFAs, but the cattle were more slow-growing and thus the meat quality was poorer (Nuernberg et al., 2005). Similar outcomes have also been found for milk production from dairy cows in high-forage systems (Dewhurst et al., 2006). If improvements could be made in forage quality, especially more high-sugar varieties as outlined above, then potentially huge improvements in the animal production can be made.

In conclusion, utilizing the information obtained from the research effort to improve grain crops and the knowledge gathered from model systems like *Arabidopsis*, offers an excellent future perspective for improving the nutritional quality and yield for forage crops.

## Author Contributions

NC and AM wrote the manuscript and conceived the perspective, read, and approved the final manuscript.

## Conflict of Interest Statement

The authors declare that the research was conducted in the absence of any commercial or financial relationships that could be construed as a potential conflict of interest.
